# Treatment of Delayed Hypersensitivity After Injection of Nasal Fillers

**DOI:** 10.1111/jocd.16681

**Published:** 2024-12-08

**Authors:** Liya Jiang, Fei Chen, Meiyang He, Yuejie Zhou, Qingqian Wei, Jintian Hu

**Affiliations:** ^1^ Department of Cosmetic Injection Center, Plastic Surgery Hospital Chinese Academy of Medical Sciences and Peking Union Medical College Beijing China; ^2^ The First Clinical College Guangzhou Medical University Guangzhou China; ^3^ The Third Clinical College Guangzhou Medical University Guangzhou China; ^4^ Xi'an Jiaotong University Xi'an Shaanxi China; ^5^ Plastic Surgery Hospital Chinese Academy of Medical Sciences and Peking Union Medical College Beijing China

**Keywords:** augmentation rhinoplasty, delayed hypersensitivity, filler injection, hypersensitivity, intralesional drug injection

## Abstract

**Background:**

Soft tissue filler injection is the second most commonly performed cosmetic procedure worldwide, with augmentation rhinoplasty being the most common filling surgery in Asian countries because Asians predominantly have a low nasal bridge. With the increasing pursuit of beauty, the adverse reactions after injection not only damage the patient's appearance but also greatly affect their quality of life. Therefore, exploring effective methods to address adverse reactions after nasal filler injections is necessary.

**Aims:**

Herein, we aimed to explore safe and effective drug doses and concentrations for the treatment of two patients who developed an allergic reaction after a nasal filling by intralesional drug injection.

**Methods:**

Two patients developed severe swelling and deformity of the nose after the injection of fillers 6 months to 1 year prior to presentation. High‐frequency ultrasound and local conditions revealed inflammation in the injected area. The patients were administered the combination of 0.7% triamcinolone acetonide, 0.42% 5‐fluorouracil, and 0.7% lidocaine into the affected area; they were followed up for 6 months.

**Results:**

After three times of treatment, swelling and sclerosis disappeared after 44.5 ± 2.12 days. No adverse effects of the infections were observed.

**Conclusions:**

This drug regimen may represent a safe and effective therapeutic strategy for the treatment of complications after soft tissue filler injections.

## 1 Introduction

Rhinoplasty addresses aesthetic and functional issues caused by nasal defects, particularly prevalent in Asian countries with naturally lower nasal bridges. Granulomatous hypersensitivity is clinically the most common type of delayed hypersensitivity; it is characterized by swelling, erythema, nodules [1]. However, nasal filler injections may lead to various complications. Regarding the classification of complications, the expert panel proposes categorizing them based on the time of onset into immediate, early, and delayed complications [2]. Early complications refer to adverse reactions occurring within 24 h after injection, whereas early delayed complications manifest from 24 h to 4 weeks postinjection. Both types commonly present with symptoms such as erythema, edema, pain, bruising, itching, and occasionally non‐fluctuant nodules, sensory abnormalities, or rare occurrences like visual disturbances [3]. Delayed complications can be classified into injection site reactions, such as redness, pruritus, and nodules, as well as infections, migration of fillers, and neurovascular injuries caused by manipulation errors [2]. As for the mechanism of delayed hypersensitivity after facial filling, Artzi et al. suggested that it may be caused by a virus infection, active sinusitis, the use of inferior products, the combination of different products or inappropriate technology [4].

## 2 Materials and Methods

This study was performed in accordance with the guiding principles of the Declaration of Helsinki and was approved by our Institutional Review Board. The patients who participated in this study provided written informed consent. To ensure the safety of patients and health care workers, all patients underwent hematologic examination before treatment to confirm that the patient had no active infectious disease, no pregnancy or lactation, stable vital signs, and conscious.

Two patients presented with delayed and severe swelling of the nasal area, which occurred a few months after the injection of soft tissue fillers. The patients underwent high‐frequency ultrasound to determine the condition of the affected area. The formula is to mix 0.7% triamcinolone acetonide with 0.42% 5‐fluorouracil and 0.7% lidocaine. The configuration method is as follows: A 3 mL solution was made using 0.5 mL 5‐fluorouracil (10 mL/250 mg), 0.5 mL triamcinolone acetonide (1 mL/40 mg), 1 mL of lidocaine, and 1 mL of saline. The affected and surrounding areas are then injected at a volume of 1 mL/1 cm^2^ using a 1 mL Luer‐lock syringe and a 30 g 25 mm needle, avoiding the epidermis. The drug regimen was the same for the three treatments, with injections every 10 to 14 days. The patients were followed up regularly for 6 months.

## 3 Results

### 3.1 Case 1

**FIGURE 1:**
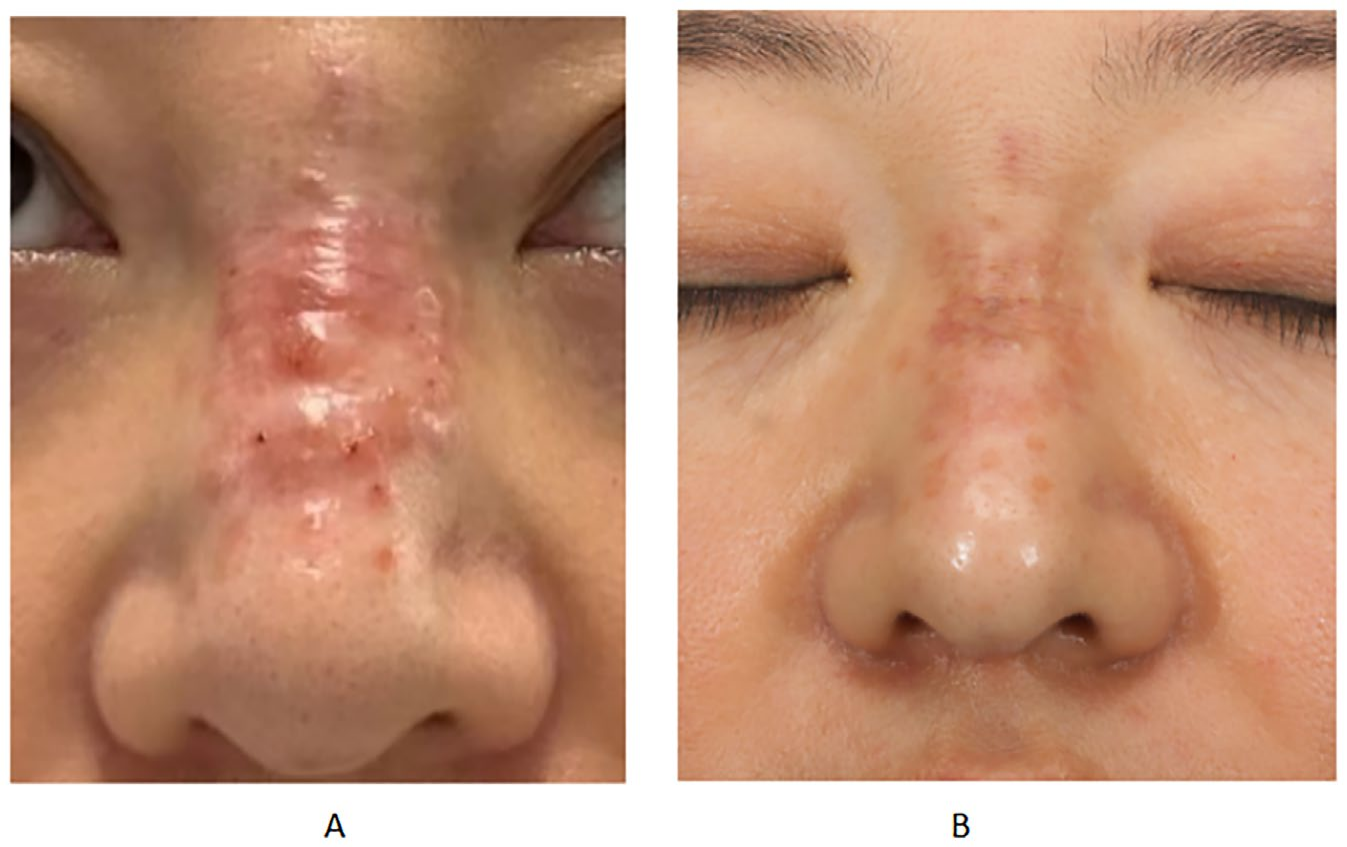
Images from the case of a 41‐year‐old woman who presented with nasal swelling following an injection of filler material. The images were 2023 in September (A) and December (B).

A 41‐year‐old woman presented to our institution with swelling of the nasal area. The patient had undergone nose augmentation through an injection of filler material at a different hospital 1 year prior to presentation. The patient contracted coronavirus disease 2019 6 months before the current presentation; 1 month after this infection, she experienced gradual swelling and hardening of the nose. An examination revealed severe swelling of the nasal area, which was hard in nature and demonstrated no mobility upon palpation. The skin of the area appeared erythematous and raised, significantly affecting the patient's facial appearance. A high‐frequency ultrasound revealed abnormal echogenicity in the subcutaneous tissue from the nasal bridge to the dorsal side, with abundant blood flow in the surrounding area, indicating inflammation associated with the filler. The patient received 2 mL of a combination of triamcinolone acetonide (13 mg) and 5‐fluorouracil (83 mg). The next two injections were given every 10–14 days. Forty‐three days after the first injection, the patient's nasal swelling basically recovered, as expected. Follow‐up at 6 months showed the patient's condition is good, with no recurrence and no complications such as hyperpigmentation, skin atrophy or indentations (Figure 1).

**FIGURE 2:**
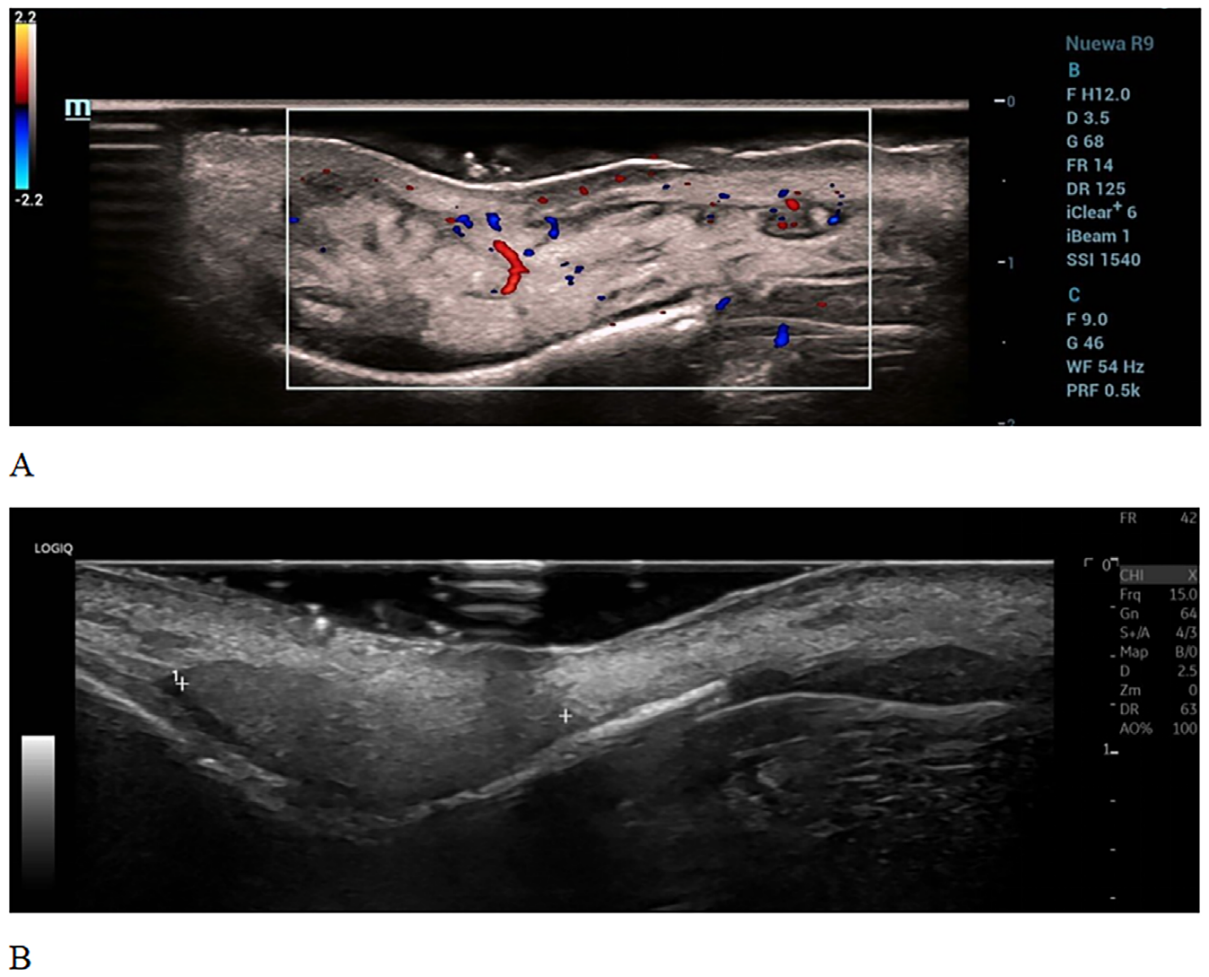
Facial color ultrasound images of Case 1. (A) Before treatment, an area of abnormal echogenicity (approximately 4.6 × 1.7 × 0.7 cm in size) is observed in the deep subcutaneous fat layer from the nasal root to the nasal dorsum. It is closely attached to the bone, with uneven internal echogenicity. A band‐like low echogenicity is visible, with point‐like blood flow seen inside and a slightly abundant blood flow visualized around it. (B) After 14 days of treatment, an area of high echogenicity (approximately 2.0 × 1.2 × 0.6 cm in size) is observed in the deep subcutaneous fat layer from the nasal root to the lower part of the nasal dorsum. It is closely attached to the bone, with moderately uniform internal echogenicity. The boundary with the surrounding tissue is partially unclear, and the band‐like boundary is weakened. It is located approximately 0.10 cm beneath the surface, mainly on the upper part of the hump. No obvious blood flow is visible inside, but a small amount of blood flow is visible around it. The abnormal area and blood flow are reduced as compared to those observed during the pretreatment examination.

A high‐frequency ultrasound examination revealed a significant reduction in abnormal echogenicity in the area, suggesting a decrease in the infection‐related swelling (Figure 2).

### 3.2 Case 2

**FIGURE 3:**
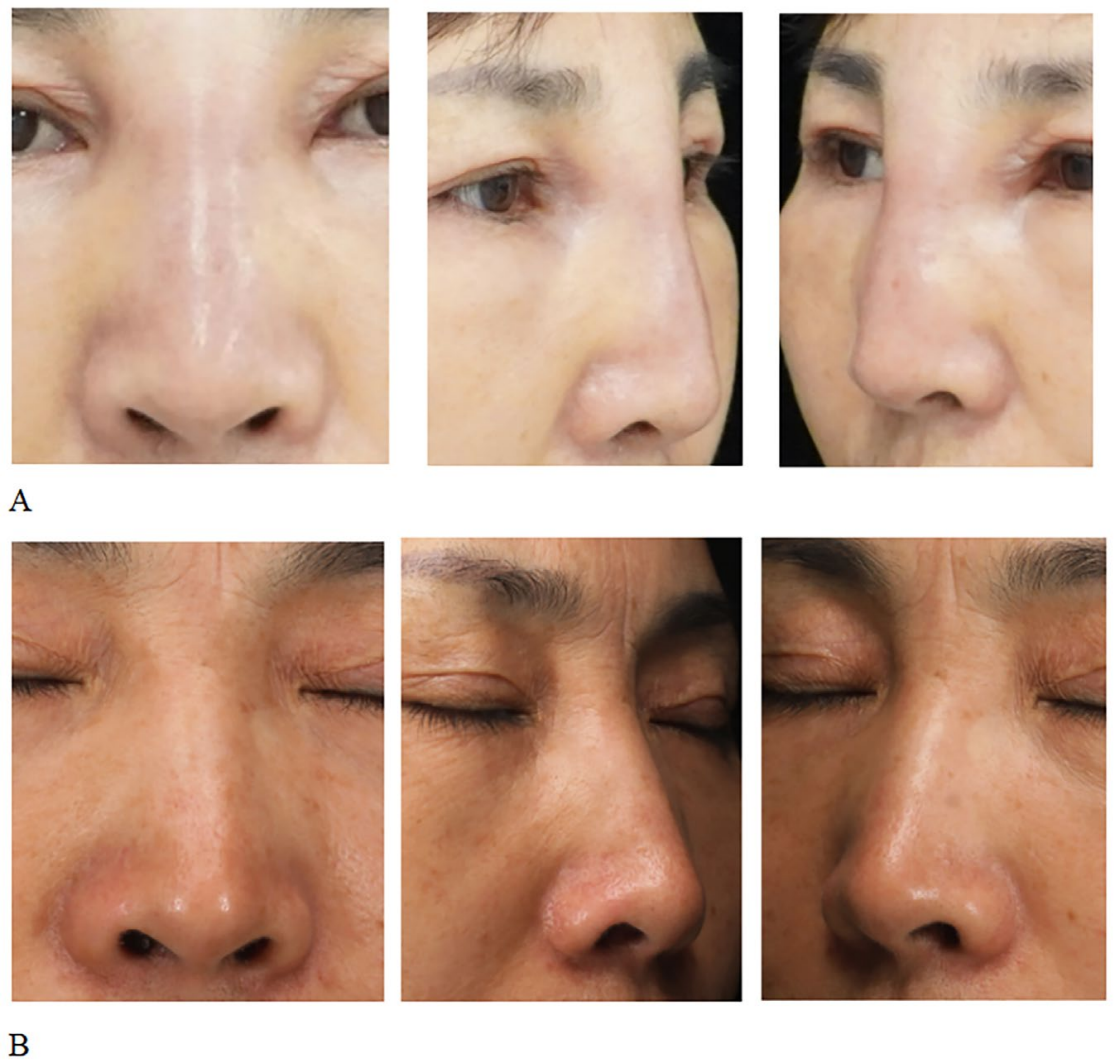
Image from a 50‐year‐old woman who presented with nasal swelling following an injection of filler material. The images were taken before (A) and 3 months after treatment (B).

A 50‐year‐old woman presented to our institution with swelling of the nasal area. The patient had undergone nose augmentation through the injection of filler material at a different hospital 6 months previously. Five months after the procedure, the patient experienced itchiness around the nasal area and gradually developed nasal redness, swelling, and deformity. An examination revealed increased tissue growth in the nasal area, which felt hard upon palpation. A high‐frequency ultrasound examination revealed thickening of the subcutaneous nasal tissue, which became mobile under pressure, and increased blood flow signals in the soft tissue; this indicated an infection. The patient received an injection of triamcinolone (13 mg) and 5‐fluorouracil (83 mg). Significant improvement was observed within only 2 days. The entire treatment cycle was expected to be 45 days, and patients completed three injections after 46 days. After the treatment, the nasal swelling disappeared and the surface returned to normal. After 6 months of follow‐up, the patients were in good condition, without recurrence, pigmentation, skin atrophy, and other complications (Figure 3).

**FIGURE 4:**
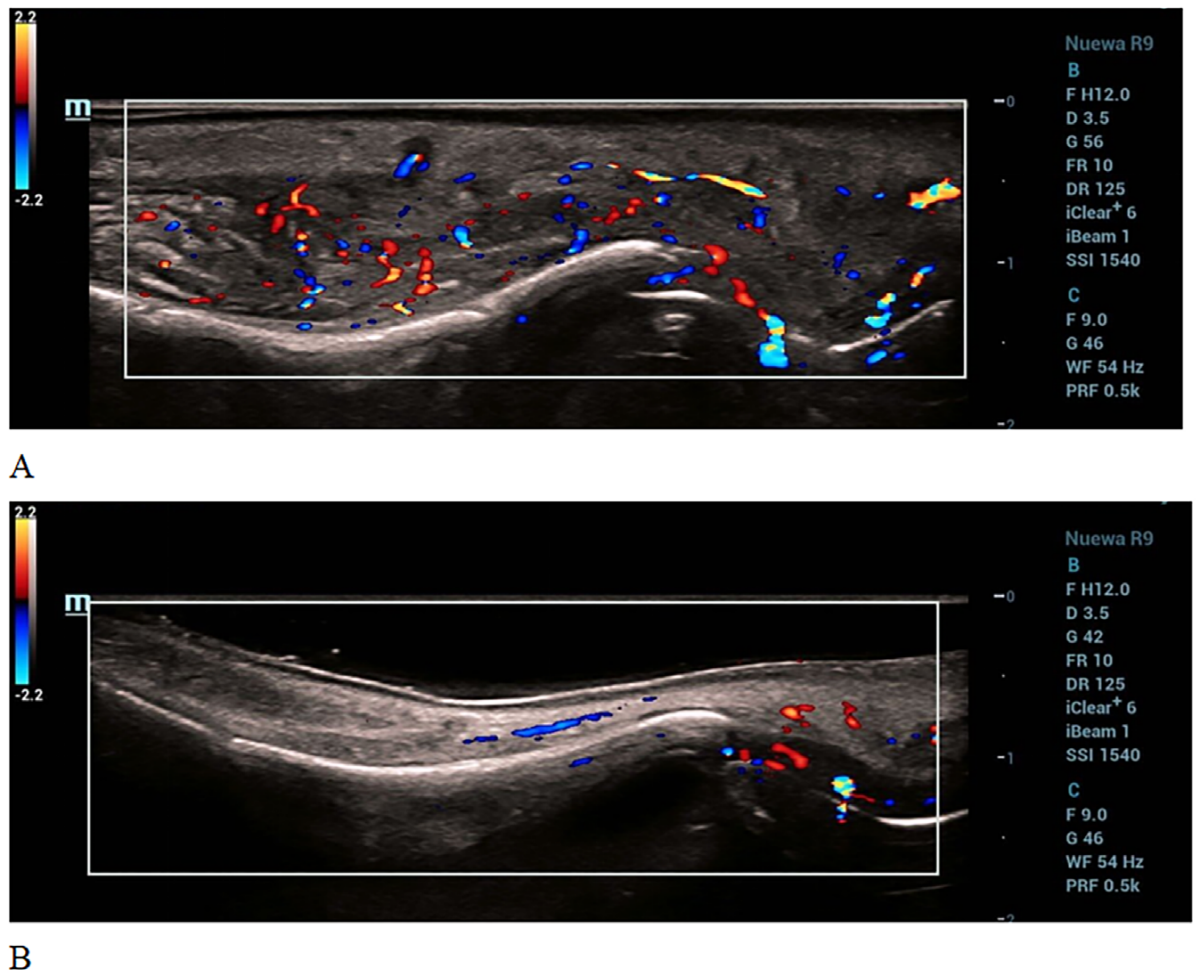
Facial color ultrasound images of Case 2. (A) Before treatment, thickening and chaotic echogenicity are observed in the subcutaneous soft tissue of the nose, mainly in the nasal root area, with a thickness of approximately 1.2 cm. The echogenicity of the deep soft tissue in this area is reduced, with the thickest part measuring approximately 0.9 cm. The surrounding soft tissue shows enhanced echogenicity. Irregular low‐echogenicity areas with unclear boundaries and fine punctate echogenicity inside are visible. Compression appears to cause floating. Abundant blood flow signals are detected in the soft tissue of the nose. (B) After 10 days of treatment, the subcutaneous structures at the root of the nose remain clear. However, the echogenicity of the subcutaneous soft tissue is poor. No obvious abnormal blood flow signals are observed.

A high‐frequency ultrasound examination revealed a reduced abnormal echo range and blood flow, with gradually clear lesion boundaries (Figure 4).

## 4 Discussion

The popularity of soft tissue filler injections has increased rapidly in recent years because they achieve good cosmetic effects without the need for surgery [3]. This increase in demand has led to the development of an increasing number of filler materials, each with its own characteristics, functions, injection requirements, and associated complications (such as vascular edema, bruising, nodules, and itching) [5]. It is necessary to develop effective therapeutic strategies to rectify these various complications. The two patients described here developed delayed‐type hypersensitivity following the injection of unidentified filler materials in the nasal area, which mainly manifested as redness, swelling, and deformity. Traditional treatment methods involve antibiotics; however, because this can be associated with a long treatment course and the patients remain susceptible to recurrence, we sought novel drug treatment that could control the condition more effectively. After three rounds of treatment, nasal swelling subsided and the skin color returned to normal. At the 6‐month follow‐up, both patients expressed satisfaction with the treatment results and reported no other issues. Therefore, the effective and safety drug regimen proposed in this report may be helpful for managing inflammation caused by injections of filler material.

Delayed immune responses, the main mechanism for defending against intracellular pathogens, are immunologically cell‐mediated immunity processes involving T cells and cytokines. It can also be a cause of physical abnormalities, including granulomatous inflammation, calcification, and casein‐like necrosis [6]. The cases in the study presented with chronic inflammation and a well‐defined nodular lesion, and were therefore suspected of granulomatous inflammation. However, due to the choice of minimally invasive treatment for injection, the diagnosis of granulomatous inflammation can not be confirmed by pathological examination. Circulating granulocytes move to soft tissue as a first line of defense, and monocytes turn into macrophages. The filler binds to toll‐like receptors to enhance innate immune responses and also enhances antigen‐presenting cell activity and cytokine expression. Failure of phagocytosis can lead to granuloma formation, and aggregation of activated macrophages can be epithelioid. Different types of giant cells and the surrounding infiltrating T lymphocytes secrete tumor necrosis factors, interferon, and Il‐12, which are responsible for sustained macrophage activation and inflammatory cell recruitment. Eventually, the granuloma is surrounded by fibrotic margins [7, 8].

The drug regimen that we adopted involved a combination of triamcinolone acetonide and 5‐fluorouracil, which was injected into the lesion and the surrounding tissues. This combination can quickly alleviate inflammation and reduce swelling. Triamcinolone acetonide is a long‐acting corticosteroid widely used for the treatment of allergic reactions, dermatological conditions, and rheumatoid arthritis. It is considered the gold standard treatment for hypertrophic scars and has excellent anti‐inflammatory effects [9]. It acts by binding and activating glucocorticoid receptors and inhibiting multiple processes, such as pro‐inflammatory cytokine production, prostaglandin and leukotriene synthesis, and arachidonic acid release; it also activates anti‐inflammatory transcription factors [10, 11]. However, triamcinolone acetonide monotherapy can result in pain, bleeding, and secondary infections; furthermore, its antiproliferative effects can inhibit fibroblast and keratinocyte formation, thereby slowing collagen synthesis and allowing collagen degradation by collagenase, leading to skin atrophy [9, 12]. Future, 5‐fluorouracil is a pyrimidine analog widely used in cancer treatment. It interferes with DNA synthesis and mRNA translation, thereby inhibiting proliferation effects and inducing apoptosis; this can, to a certain extent, suppress immune responses. However, similar to most chemotherapeutic drugs, it lacks selectivity and can cause various serious complications [13]. The widely reported adverse reactions include infusion reactions, rashes, fever, nausea, vomiting, peripheral neuropathy, and liver damage [14]. Emmerich VK et al. [15] collected data from a group of patients treated with topical 5‐fluorouracil actinic keratosis, most of whom developed skin redness, itching, rashes, and even atrophy, two patients stopped treatment because of severe reactions. Both triamcinolone acetonide and 5‐fluorouracil are prone to inducing adverse reactions when used as monotherapy. However, previous studies have shown that combining the two drugs can limit their adverse effects. Combination therapy has allowed a reduction in the required dose of triamcinolone acetonide, thereby reducing the risk of steroid‐related adverse events [16]. Jiang et al. [17] reported that compared with patients treated with either drug alone, patients treated with a combination of triamcinolone acetonide and 5‐fluorouracil for proliferative and hypertrophic scars achieved better treatment outcomes with fewer adverse effects. These results led to our selection of these two drugs for the treatment of inflammation induced by the injection of filler material.

Triamcinolone combined with 5‐fluorouracil (5‐FU) is not uncommonly used. Aguilera SB et al. successfully treated noninflammatory nodules associated with CAHA injections using a regimen of 5‐fluorouracil, dexamethasone, and triamcinolone injections [18]. Sivams et al. also reported a case of a large foreign body granuloma occurring 7 years after PMMA filler injection, which responded well to injections of a mixture of 5‐fluorouracil and triamcinolone into each granuloma [19]. The innovation of our treatment regimen lies in combining previous protocols and data, adjusting concentrations and dosages of the two drugs to significantly enhance therapeutic efficacy while ensuring higher safety.

Sivams et al. have described injecting 2.5 mL of triamcinolone (10 mg/mL) and 0.5 mL of 5‐fluorouracil (50 mg/mL) mixture into individual granulomas, totaling 4.5 mL injected volume, resulting in significant shrinkage of granulomas 1 month later [19]. Previous discussions highlighted the advantages of combining both drugs, reducing individual dosages to lower complication rates, and synergistically targeting lesions. Previous protocols mostly involved single injections with relatively high drug concentrations, increasing the risk of complications [18, 19]. Therefore, we diluted triamcinolone acetonide and 5‐FU sixfold, resulting in a significant reduction in concentration and total dose compared with previous drugs and improved safety. In contrast, the protocol had a short effective plasma concentration maintenance time and limited efficacy with a single injection, so we set the injection interval at 10–14 days and performed three injections. Such not only can ensure safety, but also according to the patient's recovery flexible adjustment of medication. Furthermore, we emphasize specific injection techniques such as deep subcutaneous injections to reduce skin atrophy and vascular dilation caused by triamcinolone, ensuring multi‐layered and uniform delivery to lesions.

And we are able to monitor early complications during the three courses of the injection, and during the 6‐month follow‐up, we ask patients to come to the hospital every month for a follow‐up and telephone follow‐up every two weeks, the goal is to observe and manage the late‐stage side effects of the 5‐fluorouracil and triamcinolone acetonide combination. There are references in the literature to late complications after repeated injections of triamcinolone acetonide and 5‐fluorouracil, the former may have skin atrophy, Telangiectasia, abnormal pigmentation, etc. In the latter, severe erythema, blisters, and even ulcers develop [20, 21]. Our patients experience no discomfort during this period, thanks to the precision of our drug regimen and injections.

## 5 Conclusion

The combination of triamcinolone acetonide and 5‐fluorouracil, when injected into areas of inflammation following soft tissue nasal filler injections, ameliorated the inflammatory response and reduced swelling in two patients in a fairly rapid manner. The drug regimen must be further investigated for the treatment of post‐filler injection complications.

## Author Contributions

Liya Jiang and Fei Chen analyzed the data and wrote the paper. Meiyang He and Yuejie Zhou performed the study. Jintian Hu and Qingqian Wei designed the study. All the authors agreed to publish the manuscript and the manuscript.

## Ethics Statement

All procedures performed in studies involving human participants were in accordance with the ethical standards of the institutional and/or national research committee and with the 1964 Helsinki Declaration and its later amendments or comparable ethical standards. This study was performed in accordance with the guiding principles of the Declaration of Helsinki and was approved by the Institutional Review Board. The two patients who participated in this study both signed informed consent forms.

## Conflicts of Interest

The authors declare no conflicts of interest.

## Data Availability

The authors have nothing to report.
